# Relaxation of Selective Constraints Causes Independent Selenoprotein Extinction in Insect Genomes

**DOI:** 10.1371/journal.pone.0002968

**Published:** 2008-08-13

**Authors:** Charles E. Chapple, Roderic Guigó

**Affiliations:** 1 Center for Genomic Regulation, Universitat Pompeu Fabra and Institut Municipal d'Investigació Mèdica, Barcelona, Catalonia, Spain; 2 Center for Genomic Regulation, Universitat Pompeu Fabra, Barcelona, Catalonia, Spain; Indiana University, United States of America

## Abstract

**Background:**

Selenoproteins are a diverse family of proteins notable for the presence of the 21st amino acid, selenocysteine. Until very recently, all metazoan genomes investigated encoded selenoproteins, and these proteins had therefore been believed to be essential for animal life. Challenging this assumption, recent comparative analyses of insect genomes have revealed that some insect genomes appear to have lost selenoprotein genes.

**Methodology/Principal Findings:**

In this paper we investigate in detail the fate of selenoproteins, and that of selenoprotein factors, in all available arthropod genomes. We use a variety of *in silico* comparative genomics approaches to look for known selenoprotein genes and factors involved in selenoprotein biosynthesis. We have found that five insect species have completely lost the ability to encode selenoproteins and that selenoprotein loss in these species, although so far confined to the Endopterygota infraclass, cannot be attributed to a single evolutionary event, but rather to multiple, independent events. Loss of selenoproteins and selenoprotein factors is usually coupled to the deletion of the entire no-longer functional genomic region, rather than to sequence degradation and consequent pseudogenisation. Such dynamics of gene extinction are consistent with the high rate of genome rearrangements observed in Drosophila. We have also found that, while many selenoprotein factors are concomitantly lost with the selenoproteins, others are present and conserved in all investigated genomes, irrespective of whether they code for selenoproteins or not, suggesting that they are involved in additional, non-selenoprotein related functions.

**Conclusions/Significance:**

Selenoproteins have been independently lost in several insect species, possibly as a consequence of the relaxation in insects of the selective constraints acting across metazoans to maintain selenoproteins. The dispensability of selenoproteins in insects may be related to the fundamental differences in antioxidant defense between these animals and other metazoans.

## Introduction

**Figure 1:**
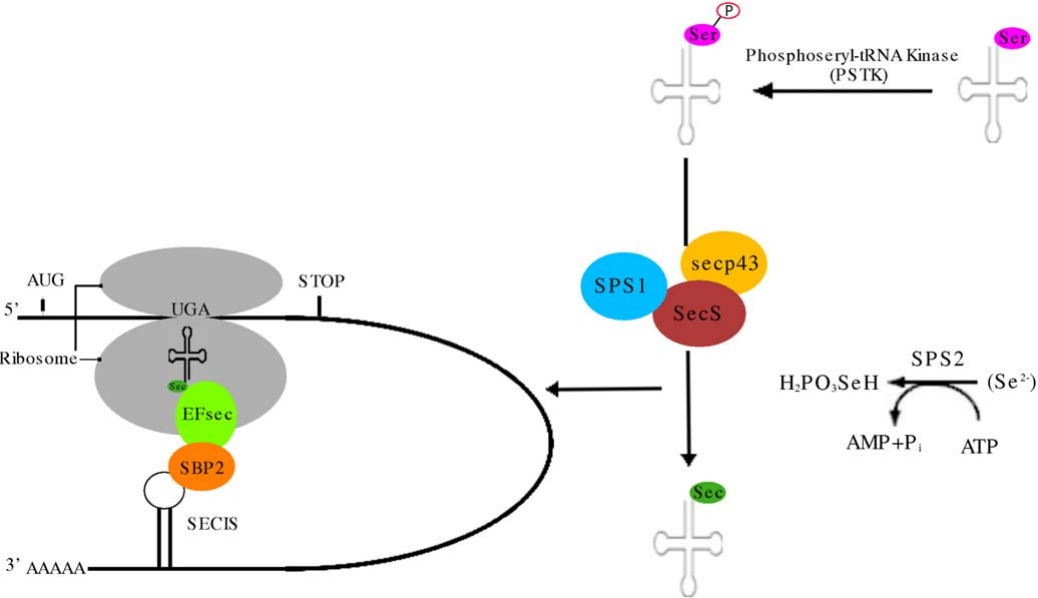
Selenocysteine biosynthesis and selenoprotein translation pathways. Selenoproteins incorporate the amino acid Selenocysteine (Sec) which is coded by the codon UGA, normally a stop codon. The recoding of UGA as a Sec codon is mediated by a structural element on the 3′ Untranslated Region (UTR) of selenoprotein mRNAs, the SElenoCysteine Insertion Sequence (SECIS). This is recognised by the SECIS Binding Protein 2 (SBP2), which binds to both the SECIS element and the ribosome. SBP2, in turn, recruits the Sec-specific Elongation Factor EFsec, and the selenocysteine transfer RNA, tRNA^Sec^. SBP2 and tRNA^Sec^ form a complex with the tRNA Selenocysteine associated protein, secp43. Sec is synthesized from serine in a multi-step reaction: Ser-tRNA^[Sec]^ is phosphorylated by A Phosphoseryl tRNA Kinase (PSTK) and converted to Sec-tRNA^[Sec]^ by Sec synthetase (SecS). Secp43 is also known to be involved in the conversion from seryl to selenocysteyl but its exact role is unclear. Finally, Selenophosphate Synthetase 2 (SPS2), catalyses the formation of mono-selenophosphate, the donor compound of Selenium necessary for the synthesis of selenocysteine, from either selenite (SeO_3_) or from an unstable selenide compound depicted as (Se^2−^). The exact role of SPS1 is still not clear. This figure was partially adapted from [7].

Selenoproteins are a diverse family of proteins containing Selenium (Se) in the form of the non-canonical amino acid selenocysteine (Sec). Selenocysteine, the 21st amino acid, is similar to cysteine (Cys) but with Se replacing Sulphur. In many cases the homologous gene of a known selenoprotein is present with cysteine in the place of Sec in a different genome. Selenocysteine is coded by the opal STOP codon (TGA). Since this codon normally signifies an end to translation, a number of factors combine to achieve the co-translational recoding of TGA to Sec (Figure 1). The 3′ UTRs of selenoprotein transcripts contain a stem-loop structure called a **SE**leno**C**ysteine **I**nsertion **S**equence (SECIS) element. This is recognised by the **S**ECIS **B**inding **P**rotein 2 (SBP2), which binds to both the SECIS element and the ribosome. SBP2, in turn, recruits the Sec-specific Elongation Factor EFsec, and the selenocysteine transfer RNA, tRNA^Sec^. SBP2 and tRNA^Sec^ form a complex with the tRNA **Se**leno**c**ysteine associated **p**rotein, secp43, which is believed to be involved in the regulation of selenoprotein translation [1]. Ribosomal protein L30 has recently been shown to interact with the SECIS element and compete with SBP2 for SECIS binding in a Magnesium dependent manner [2].

Unlike most amino acids, which are aminoacylated onto their cognate tRNAs, Sec is synthesized from serine in a multi-step reaction while bound to its unique tRNA^[Ser]Sec^ [3]. Although this reaction is well understood in prokaryotes (e.g. [4]), the details of the eukaryotic pathway remain elusive. It has recently been demonstrated that the protein previously known as **S**oluble **L**iver **A**ntigen/**L**iver **P**ancreas antigen (SLA/LP) is the eukaryotic homolog of bacterial Sec synthetase (SecS), and converts the seryl-tRNA^[Ser]Sec^ to selenocysteil-tRNA^[Ser]Sec^ [5]. A **P**hospho**s**eryl **t**RNA **K**inase (PSTK) has also been identified and shown to convert seryl-tRNA to phosphoseryl-tRNA, a likely intermediate to selenocysteil-tRNA [6]. Finally, Selenophosphate Synthetase 1 and 2 (SPS1 and SPS2), which exhibit sequence similarity, catalyse the formation of mono-selenophosphate, the donor compound of Selenium necessary for the synthesis of selenocysteine. A summary of the selenocysteine biosynthesis and selenoprotein transcription pathways can be seen in Figure 1. Interestingly, SPS2 is itself a selenoprotein. Since selenocysteine, and therefore mono-selenophosphate, is necessary for the expression of SPS2, it has been suggested that SPS1 manufactures basal levels of this compound and the more reactive SPS2 takes over under stimulatory conditions [7].

Selenoproteins exist in all domains of life, Eukarya, Eubacteria and Archaea. However, no selenoproteins have been found in higher plants (one has been identified in the green alga *Chlamydomonas reinhardtii*) [8] or fungi. Vertebrate genomes encode up to 25 selenoprotein genes [9], [10], while invertebrate genomes encode fewer [11], [12]. Three selenoprotein genes have been found in *D. melanogaster* [12], *SPS2*, *SelH* and *SelK*. SPS2 is involved in selenoprotein biosynthesis (see above), while SelH and SelK are poorly characterized functionally, but they seem to play an antioxidant role [13], [14]. It has been reported that inhibiting either SelK or SelH expression significantly reduces viability in embryos [15]. Both SelK and SelH have Cys-paralogs in the *D. melanogaster* genome.

Remarkably, only one selenoprotein (Thioredoxin reductase) has been identified in the *C. elegans* genome [11]. That the entire machinery of selenoprotein synthesis has been conserved in *C. elegans* for synthesizing a single protein had been taken, until very recently, as an indication that selenoproteins are essential for animal life. Indeed, mouse tRNA^Sec^ knock-outs have been shown to be lethal in-utero [16]. Similarly, mutant flies for SPS1 do not contain selenoproteins and are lethal at third instar larvae [17]. In contrast, Hirosawa-Takamori et al [18] have reported that mutant flies for EFsec also fail to decode TGA as Sec but are viable and fertile.

Recently, we have shown [19] that one fly, *Drosophila willistoni*, lacks selenoprotein genes, being the first animal reported to lack these proteins. More recently Lobanov et al. have reported that other insect genomes also appear to lack selenoproteins [20]. In this paper, we extend these results by performing an exhaustive analysis of all available arthropod genomic sequences searching for selenoproteins and selenoprotein factors.

First, we analyzed the genomes of the 12 Drosophila species recently sequenced [19]. In addition to the fact that in *D. willistoni* two of the known insect selenoproteins (SelH and SelK) are Cys-homologs, while the third (SPS2) appears to have been lost [19], we have found that many of the genes involved in selenoprotein synthesis have been lost in *D. willistoni*, including the tRNA specific for Sec (tRNA^Sec^). This is strongly indicative that *D. willistoni* not only lacks the *D. melanogaster* selenoprotein reference complement but that it has lost the ability to synthesize selenoproteins altogether. However, other genes thought to be involved in selenoprotein synthesis are as conserved in *D. willistoni* as in the other Drosophila genomes, suggesting that these proteins are involved in additional pathways other than selenoprotein synthesis. Overall, our analyses show selenoprotein evolution in Drosophila to be a very dynamic process; other deviations from the reference selenoprotein complement include the loss of SelK as a selenoprotein in *D. persimilis* and the duplication of SelH in *D. grimshawi*.

We have also analyzed the sequences of all other available insect genomes (the mosquitoes *Anopheles gambiae* and *Aedes aegypti*, the honey bee *Apis mellifera*, the wasp *Nasonia vitripennis*, the beetle *Tribolium castaneum* and the silkworm *Bombyx mori*), and found that, while mosquitoes share the selenoprotein complement of *D. melanogaster*, selenoproteins have been lost in the wasp, the honey bee, the silkworm and the beetle. Analysis of available sequence data from other arthropoda (including cDNA, EST, protein and genomic data) suggests that the loss of selenoproteins has been confined to the infraclass Endopterygota, affecting species of all orders investigated (Hymenoptera, Lepidoptera, Diptera and Coleoptera). Interestingly, however, it is not possible to identify a single evolutionary event leading to the loss of selenoproteins in all these species. That most known Diptera still conserve selenoproteins and the mosaic pattern of selenoprotein loss in the other species suggest, instead, multiple independent events of selenoprotein loss in insects. This pattern of gene loss is consistent with a relaxation of the selective constraints acting on insects to maintain selenoproteins, which could be related to the differences in antioxidant defense systems between insects and other metazoans.

## Methods

### Accession Numbers

The accession numbers for each of the *D. melanogaster* genes used in this study are as follows: SelK : [GenBank:AAF48111.2]; SelH : [GenBank:AAF48293.3]; SPS2 : [GenBank:AAN10746.2]; SPS1 :[GenBank:AAM70998.1]; SBP2 : [GenBank:AAF50448.2]; EFsec: [GenBank:AAF46721.1]; Secp43 : [GenBank:AAL90383.1]; SecS : [GenBank:AAS65099.1]; PSTK : [GenBank:AAF48985.2]; and tRNA^Sec^ : [FLYBASE:FBgn0011987]. The sequences of the other eukaryotic selenoproteins used can be found at http://genome.imim.es/datasets/2007selenoinsects/#1.

### Genome Sequence Data

The genomes of the Drosophila species were downloaded from the Drosophila Sequencing Consortium wiki (http://rana.lbl.gov/drosophila/caf1.html), we used the Comparative Analysis Freeze 1 (CAF1). The species are: *Drosophila ananassae, Drosophila erecta, Drosophila grimshawi, Drosophila melanogaster, Drosophila mojavensis, Drosophila persimilis, Drosophila pseudoobscura, Drosophila sechellia, Drosophila simulans, Drosophila virilis, Drosophila willistoni* and *Drosophila yakuba.*

The *A. mellifera* genome [21] (apiMel2, January 2005) was downloaded from UCSC, ftp://hgdownload.cse.ucsc.edu/goldenPath/apiMel2/

The *T. castaneum* [22] (release 1.1, April 2006) sequences were downloaded from NCBI, ftp://ftp.ncbi.nih.gov/genomes/Tribolium_castaneum/

The *A. gambiae* sequences [23] (anoGam1, February 2003) were downloaded from UCSC, ftp://hgdownload.cse.ucsc.edu/goldenPath/anoGam1/

The *A. aegypti* sequences [24] (AaegL1, March 2006) were downloaded from VectorBase, ftp://ftp.vectorbase.org/public_data/organism_data/aaegypti/

The *N. vitripennis* sequences (Nas1.0, March 8, 2007) were downloaded from the Human Genome Sequencing Center at the Baylor College of Medicine, http://www.hgsc.bcm.tmc.edu/projects/nasonia/

The *B. mori* sequences [25] (release 1, October 2003) were downloaded from SilkDB, http://silkworm.genomics.org.cn/silkworm/

The *D. pulex* sequences were produced by the US Department of Energy Joint Genome Institute (http://www.jgi.doe.gov/) in collaboration with the Daphnia Genomics Consortium (http://daphnia.cgb.indiana.edu).

The sequences of all other species investigated were downloaded using the NCBI ENTREZ data retrieval service, http://www.ncbi.nlm.nih.gov/gquery/gquery.fcgi.

### Selenoprotein search in insect genomes

The sequences of known selenoproteins— *D. melanogaster* when available, human when not— (see http://genome.imim.es/datasets/2008selenoinsects/#1) were searched using the program TBLASTN [26] against the genomic sequences of each investigated species. The resulting regions of high similarity (see http://genome.imim.es/datasets/2008selenoinsects/#2) were then extracted from the target genome, and specifically aligned to the query selenoprotein amino acid sequence using the genewise [27] and exonerate [28] programs with default parameters. The output of these programs was manually analyzed to build the exonic structure and the amino acid sequence of the predicted selenoprotein in the target genome. Selenoprotein genes were also searched in cDNA, EST and protein sequences when available for the investigated species.

We also investigated all arthropods with sufficient sequence data available (at least 100 genomic, EST, or peptide sequences) for the presence of the known eukaryotic selenoproteins. These were: Amblyomma americanum, Anoplophora glabripennis, Antheraea pernyi, Bactrocera dorsalis, Bactrocera oleae, Bemisia tabaci, Bombyx mandarina, Ceratitis capitata, Daphnia Pulex, Haematobia irritans, Laupala kohalensis, Acyrthosiphon pisum, Homalodisca coagulata, Ixodes scapularis, Locusta migratoria, Nasonia giraulti, Ostrinia furnacalis, Ostrinia nubilalis, Pediculus humanus, Pyrocoelia rufa, Reticulitermes flavipes, Schizaphis graminum, Thermobia domestica, and Triatoma dimidiata.

### Prediction of tRNA^Sec^

tRNAScanSe [29] was used to scan each genome for the presence of a tRNA^Sec^ gene first with default parameters and then, if no selenocysteine tRNA was found, using only Cove analysis (-C option) which increases the sensitivity. tRNAScanSe uses three models for tRNA^Sec^: Sec(e), Sec(p) and Sec. Sec(e) matches a selenocysteine model based specifically on eukaryotic tRNAs, Sec(p) matches a selenocysteine model based specifically on prokaryotic tRNAs and SeC means that the anticodon identified is UCA, but the predicted tRNA does not match specific SeC models (Lowe T.M., pers. comm.).

It must be stressed that tRNAScanSe models for tRNA^Sec^ are not as trustworthy as those for other tRNAs due to the small number of tRNA^Sec^ sequences available. tRNAScanSe fails to predict a tRNA^Sec^ in at least one species (*Takifugu rubripes)* known to code for selenoproteins (Chapple C.E. unpublished data). Therefore the lack of a tRNA^Sec^ prediction, although indicative, is not conclusive evidence for the absence of said gene in a given genome.

### Multiple Alignments of selenoprotein genes

The alignments of the amino acid sequences of selenoproteins, selenoprotein cys-homologs and selenoprotein factors were obtained using a combination of the programs clustalw [30], t_coffee [31] and mafft [32]. Where necessary, the alignments were manually edited using SEAVIEW [33]. The alignment images presented here were created by jalview [34].

### Phylogenetic trees

Phylogenetic trees were built using the online service phylogeny.fr [35] (“Advanced” Mode, no multiple alignment, all else default) which implements PhyML [36] for the construction of phylogenetic trees and treedyn [37] for producing the images presented here.

### Syntenic Alignments

For the Drosophila species, we built the syntenic regions for each of the three selenoprotein genes and each selenoprotein factor. For this we used the annotations produced by the Drosophila Sequencing Consortium [19]. We selected the 20 surrounding genes of each selenoprotein gene in *D. melanogaster*. We then checked the position of each of these on the target genome. If a gene was annotated as being on the same sequence (scaffold or chromosome depending on the genome) as the target selenoprotein, it was designated “found” and if not, “missing”. For some *D. melanogaster* genes, the target genome had no annotated homolog. In these cases the *D. melanogaster* gene was searched against the target genome using TBLASTN, and the resulting HSP was extended to the full-length protein by genewise and/or exonerate. The distance between the genes was not taken into account, only the order in which they were found. We also built syntenic regions using the whole genome multi-species alignments produced by Lior Pachter's group at UC Berkeley [19]. For each of the insect selenoproteins and selenoprotein factors, we extracted the region containing the gene in question and the immediately adjacent genes both upstream and downstream.

Although we attempted to do the same for the other insects investigated, synteny between them was not sufficient and we were unable to build the necessary alignments.

### Search for novel selenoproteins in *D. willistoni*

A modified version of the gene prediction software geneid [38] capable of predicting selenoprotein genes was run on the *D. willistoni* genome. This method has already been described in [12]. Briefly, it consists of predicting all possible SECIS elements in the target genome then running geneid with the position of these elements given as external information. Geneid will only predict a TGA-containing gene if a SECIS element is found at a suitable distance downstream. The resulting predictions are usually compared against the protein and EST non-redundant sequence databases, as well as against other genome sequences, in search of supporting evidence in the form significant alignments including the aligned Sec-Sec or Sec-Cys.

We also searched for all possible TGA-containing open reading frames (ORFs) in the *D. willistoni* genome. This approach is described in full in Taskov et al [11]. In summary, all TGA-containing ORFs, defined as genomic sequences between two non-TGA stop codons with at least one in-frame TGA and no other in-frame stop codons, are searched in the genome of interest; the resulting sequences are translated in the appropriate frame and compared against the non-redundant protein and EST databases, as well as against other genomes (of insects, in this case). Query sequences where the in-frame TGA is shown to align to either another TGA in the target sequence or to a cysteine residue, and which show conservation extending past the TGA are kept as candidates and further analyzed for the presence of SECIS elements.

### SECIS prediction

The SECIS elements in this paper were predicted using SECISearch [9], which can predict potential SECIS elements as well as assess their thermodynamic stability. Three different patterns of decreasing strictness were used allowing us to find both standard and non-standard SECIS elements (see http://genome.imim.es/datasets/2008selenoinsects/#3.)

## Results

### Loss of selenoproteins in *D. willistoni*

The three known *D. melanogaster* selenoproteins (SelK, SelH, and SPS2) are found as selenoproteins in all Drosophila genomes except *Drosophila persimilis* and *D. willistoni*. SelK is not a selenoprotein in *D. persimilis*, while in *D. willistoni* SelK and SelH are Cys-homologs, and SPS2 appears to have been lost.

### SelH

**Figure 2:**
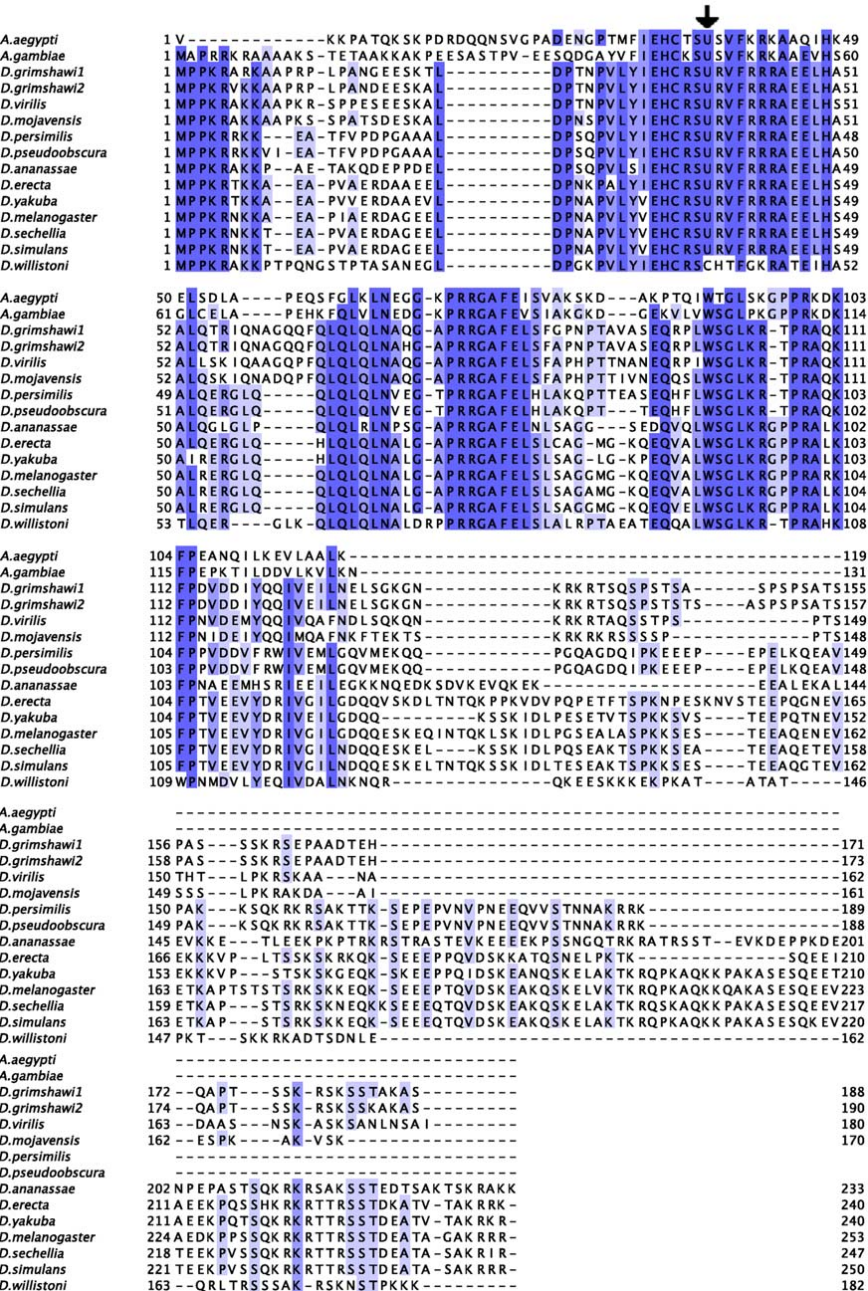
Alignment of insect SelH proteins. The black arrow shows the position of the selenocysteine (U) residue (cysteine in *D. willistoni*). Here, as in the other alignments, only insect species encoding SelH are shown.

As can be seen in Figure 2, SelH appears to be as conserved in *D. willistoni* as in the other Drosophila genomes. However, a number of residues around the Cys/Sec, conserved across all Drosophila (and other Diptera) are mutated in *D. willistoni*, suggesting adaptive changes to compensate the change from Sec to Cys, thereby maintaining the function of SelH. Such compensatory changes have been reported for thioredoxin reductases [39]. The *SelH* SECIS element, strongly conserved across Drosophila species (Figure S1), cannot be found in *D. willistoni*, nor can any alternative SECIS element. Interestingly, SelH has been duplicated in *Drosophila grimshawi*, where we found two distinct *SelH* selenoprotein genes (Figure 2).

### SelK

**Figure 3:**
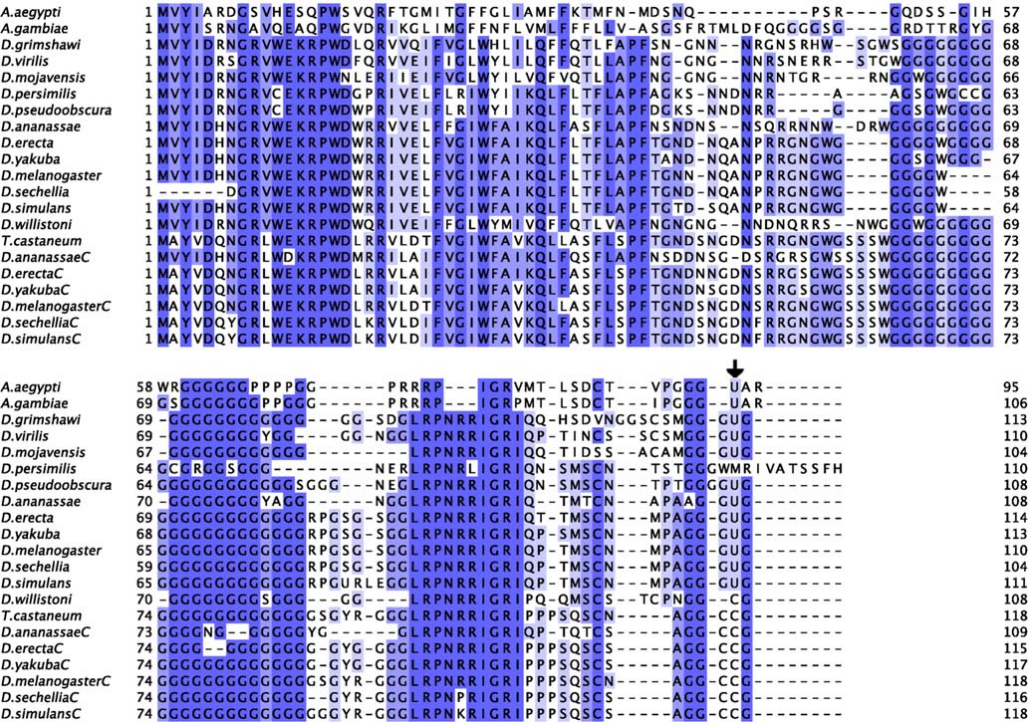
Alignment of insect SelK and SelK cysteine paralogs. SelK has been duplicated, producing a Cys-paralog, in species of the melanogaster group. These paralogs are shown with a “C” after the name of the species. The black arrow shows the position of the selenocysteine (U) residue (cysteine in *D. Willistoni* SelK and in the SelK Cys-paralogs).

With the exception of the Cys to Sec change, SelK is as conserved in *D. willistoni* as in the other Drosophila species (Figure 3). The *D. melanogaster* SelK Cys-paralog (CG1840) is only present in the melanogaster group (*D. simulans, D. sechellia, D. melanogaster, D. yakuba, D. erecta* and *D. ananassae*) and so is missing in *D. willistoni*. Indeed, the phylogenetic tree including the SelK and SelK Cys-paralogs in the 12 Drosophila species clearly shows that despite the absence of a Sec residue, *D. willistoni* SelK clusters with the selenoproteins and not the cysteine paralogs (Figure S2). Interestingly, the *SelK* SECIS element, strongly conserved across selenoprotein containing Drosophila, can still be recognized, although degenerate, in the genome of *D. willistoni* (Figure S3).

SelK is not a selenoprotein in *D. persimilis* either (Figure 3). In a previously unreported selenoprotein disabling event, the insertion of a T nucleotide has caused a frameshift, eliminating the in-frame TGA and the subsequent STOP codon, adding nine codons downstream to the next STOP (Figure S4). Consistent with the disabling mutation, the *SelK* SECIS is degenerate in *D. persimilis* (Figure S3)

### SPS2

**Figure 4:**
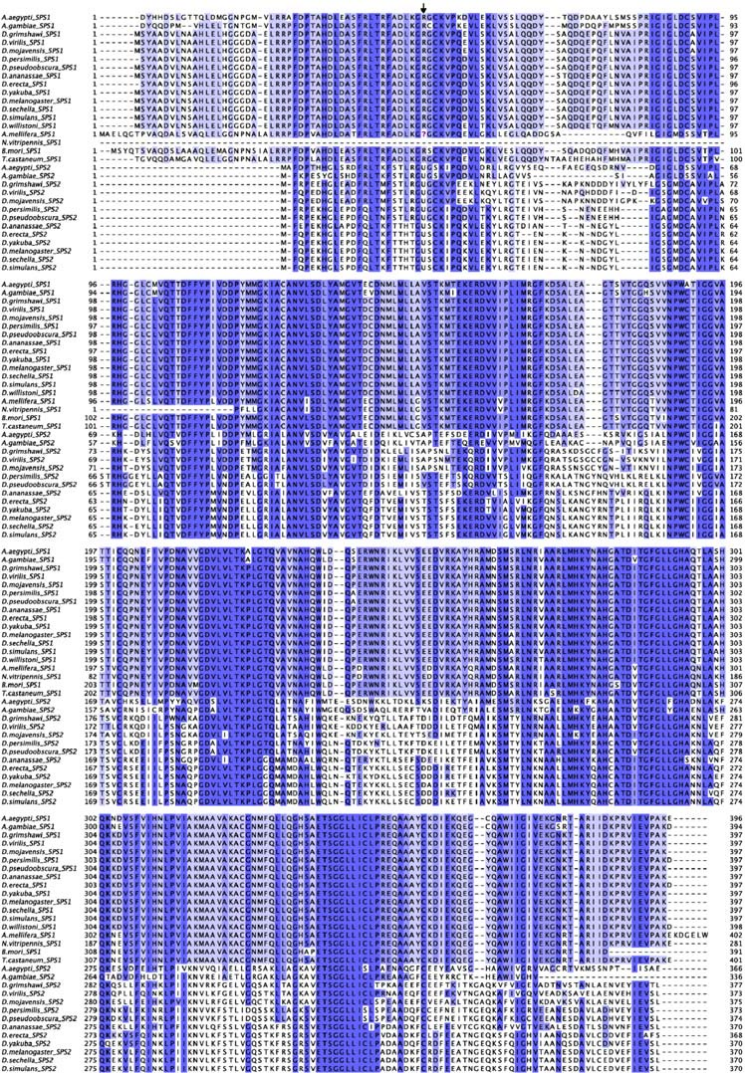
Alignment of insect SPS1 and SPS2 proteins. The black arrow shows the position of the selenocysteine residue in SPS2 and Arganine or Cysteine in SPS1. In *A. mellifera*, we use “?” to denote the codon TGA. Although we believe that in this case, TGA is being readthrough to incorporate Arginine (R), it actually aligns with the Sec-incorporating codon in SPS2.

**Figure 5:**
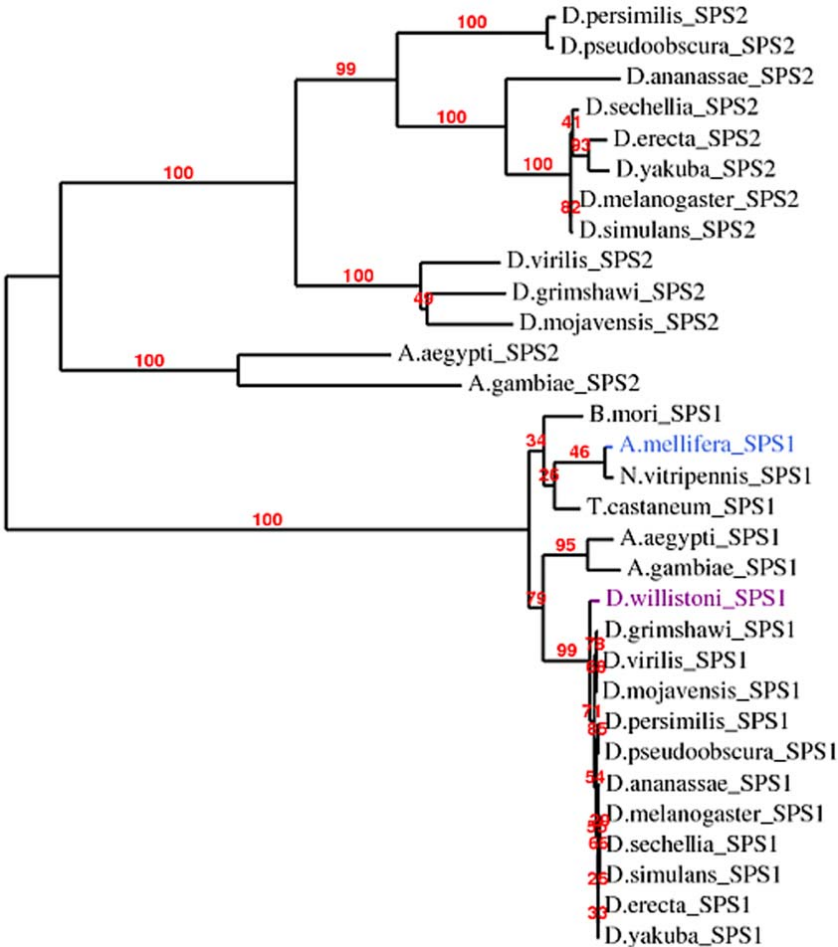
Phylogenetic tree for the insect SPS1 and SPS2 proteins. This tree was built from the alignment of all insects sequences in Figure 4. Note that the D. willistoni sequence (in magenta) clusters with the other SPS1 sequences. This is also the case of the sequence from *A. mellifera* (in blue), in spite of the fact that the in-frame UGA codon in this sequence aligns with the Sec codon in the insect SPS2 sequences.

SPS2 appears to have been lost in *D. willistoni*. Indeed, the *D. melanogaster SPS1* and *SPS2* map to the same location in the *D. willistoni* genome, but analysis of the sequence alignments (Figure 4) clearly reveals that the *D. willistoni* gene is the SPS1 homolog, as confirmed by the tree built from the multiple alignment of insect SPS1 and SPS2 proteins (Figure 5). We could not find a secondary match for the *D. melanogaster* SPS2 in *D. willistoni*, suggesting that this protein is lost in this species.

The above analyses strongly indicate that none of the known *D. melanogaster* selenoproteins is a selenoprotein in *D. willistoni*. From these analyses, however, we cannot conclude that *D. willistoni* lacks selenoprotein genes, since other selenoproteins not in *D. melanogaster* could be present in *D. willistoni*. However, we think this is highly unlikely. First, we have compared all known eukaryotic selenoproteins against the *D. willistoni* genome and have found the Cys-homologs typically found in *D. melanogaster* (15-kDa, Glutathione peroxidase (GPx), *thioredoxin* reductase (TR) and SelR). Second, we ran a modified version of the gene predictor geneid [38] capable of predicting selenoprotein genes and, in addition, we searched for all possible TGA-containing exons in the genome of *D. willistoni* (see Methods). However, after screening the predictions made by these two methods for conservation across the predicted Sec-encoding TGA and potential SECIS elements, all candidates were discarded. The strongest evidence that *D. willistoni* not only lacks selenoprotein genes, but also the capacity to synthesize selenoproteins, comes however from the analysis of the genes involved in selenoprotein biosynthesis. Indeed, that *SPS2* is lost in *D. willistoni* already indicates that selenoprotein synthesis is strongly compromised. Arguably, *SPS1*, present in *D. willistoni* (see below), could rescue SPS2 function. It has been demonstrated however, that selenoprotein biosynthesis is severely impaired in SPS2 knockdown NIH3T3 cells, and that transfection of SPS1 does not restore selenoprotein biosynthesis, suggesting that SPS1 does not complement SPS2 function [40]. Our analyses indicate, moreover, that not only SPS2, but also other crucial components of the selenoprotein biosynthesis machinery have also been lost in *D. willistoni*.

Below we describe our results for each of the factors known to be involved in selenoprotein biosynthesis. We will not focus on ribosomal protein L30 because, as an ubiquitous component of the ribosome, it was present in all species investigated and SECIS binding does not appear to be its primary function.

### tRNA^Sec^

**Figure 6:**
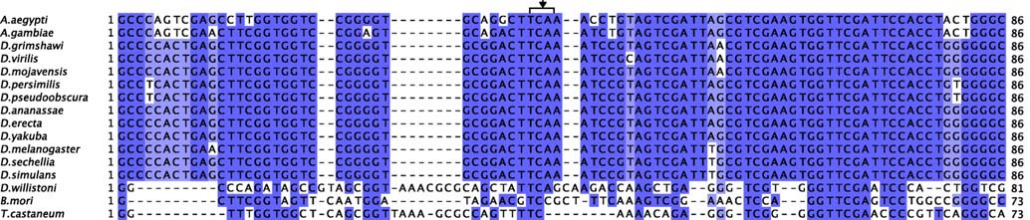
Alignment of insect tRNA^Sec^ sequences. The black arrow points to the position of the TCA anticodon.

We used tRNAScanSe to predict tRNA^Sec^ genes in each Drosophila genome (see Methods). No suitable tRNA^Sec^ could be found in the genome of *D. willistoni*, but high scoring candidates were found in all other Drosophila species (the score of the only tRNA^Sec^ prediction in *D. willistoni* was 23.16, while those of the other drosophila ranged from 50.88 to 56.88, see Table 1). Moreover, the *D. willistoni* prediction was clearly less conserved than those in the other Diptera (Figure 6), and it did not map to the syntenic region of this gene in the other Drosophila genomes. Instead, the syntenic region in *D. willistoni* shows a deletion spanning the tRNA^Sec^ locus (see http://genome.imim.es/datasets/2008selenoinsects/#4). The upstream (CG7754) and the downstream (CG12384) immediately adjacent genes are present. These data strongly suggest that *D. willistoni* has lost tRNA^Sec^.

**Table 1:** tRNA^Sec^ predictions and their scores in each species.

	tRNA^Sec^
*Drosophila*	50.88–56.88, Sec(e)
***D. willistoni***	**23.16 (Sec)**
*A. gambiae*	32.53, Sec(p)
*A. aegypti*	53.89, Sec(e)
***A. mellifera***	NA
***N. vitripennis***	NA
***B. mori***	**55.07, Sec**
***T. castaneum***	**45.28, Sec(e)**

Species lacking selenoproteins are shown in **bold.** “Sec(e)” matches a model based specifically on eukarotic tRNAs, “Sec(p)” matches a model based specifically on prokaryotic tRNAs and “SeC” means the anticodon identified is TCA, but does not match any specific SeC models (these are much less certain, and could be due to problems in correctly locating the tRNA anticodon)

### EfSec

*EFsec* was found to be highly conserved in the genomes of all the Drosophila species (Figure S5), but absent in *D. willistoni*. The best candidate found in *D. willistoni* was in fact the gene *EFtau*. No residual (pseudogenised) sequence was found when investigating the syntenic region. Instead, *D. willistoni* shows a gap spanning the *EFsec* locus. Both the upstream (CG10795) and the downstream (CG9707) immediately adjacent genes are present (see http://genome.imim.es/datasets/2008selenoinsects/#4).

### SecS

Although conserved in the other Drosophila (Figure S6), no *SecS* homolog was found in the *D. willistoni* genome. Analysis of the syntenic region across the Drosophila genomes reveals, however, that while the gene upstream of *SecS* (CG2922) is strongly conserved in the Drosophila genomes (including that of *D. willistoni*), a huge deletion, eliminating *SecS* and the gene downstream (CG2919), is present in the genome of *D. willistoni* (see http://genome.imim.es/datasets/2008selenoinsects/#4).

### pstk

*pstk* was also found to be missing in the *D. willistoni* genome and present in the other Drosophila (Figure S7). However, detailed analysis of the syntenic region in *D. willistoni* of the *D. melanogaster pstk* locus revealed a sequence that could be considered a pseudogenised pstk (see http://genome.imim.es/datasets/2008selenoinsects/#4).

### SBP2

**Figure 7:**
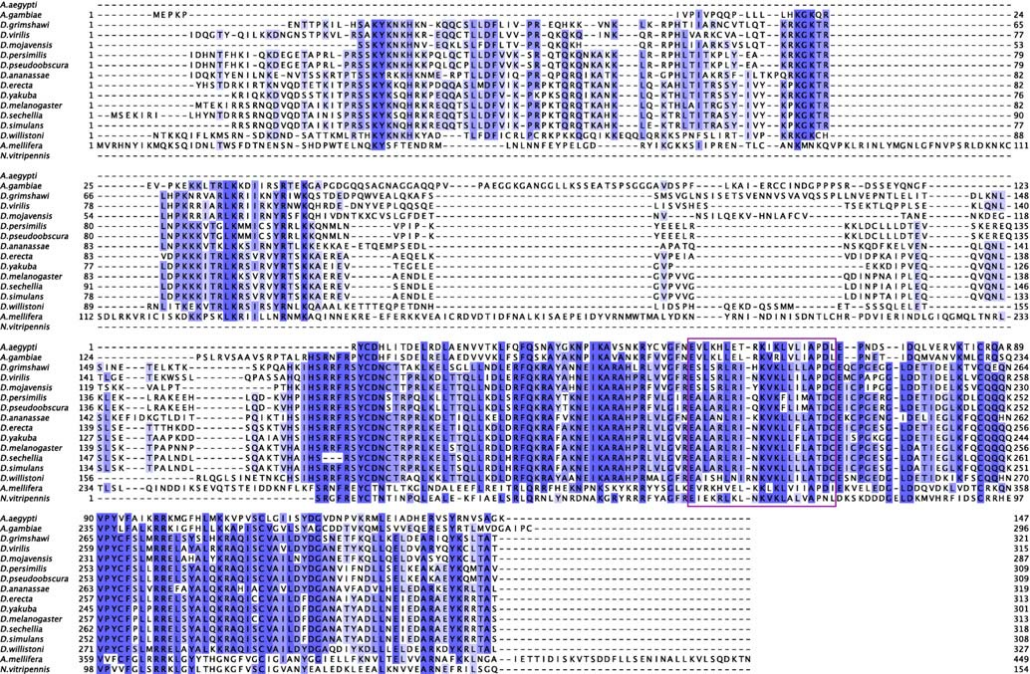
Alignment of insect SBP2 proteins. Alignment of insect SBP2 proteins. The conserved region containing the insertion in *D. willistoni* is bound by a magenta box.

An SBP2 homolog can be found in *D. willistoni* (Figure 7), in the expected syntenic region. The C-terminal region, strongly conserved across the drosophila, is also conserved in *D. willistoni*indicating that the lack of selenoproteins has not relaxed the selective constraints acting on this region of the protein. This region, however, contains the SBP2 SECIS binding domain. Within this domain a region of 19 amino acids bounded by two Glutamic Acid (E) or Aspartic Acid (D) residues has been shown to be essential for SECIS recognition. Indeed, the specific distance between these two amino acids seems to be a defining feature of the SECIS binding capacity of SBP2 ([41], Krol A., pers. comm.). Interestingly, this region in *D. willistoni* has an insertion, which could impair SECIS binding capacity (Figure 7). The N-terminal region is less conserved overall, but is particularly degenerated in *D. willistoni*.

### SPS1

Although SPS1 is present and highly conserved in *D. willistoni* (Figure 4), the phylogenetic tree derived from this protein's amino acid sequence places *D. willistoni* as sister group to the rest of the Drosophila (Figure 5), suggesting a relative acceleration of the rate of evolutionary change in this gene.

### Secp43

*Secp43* was found to be present and highly conserved in all 12 fly genomes, including *D. willistoni* (Figure S8).

In summary, our analyses reveal three different modes of evolution for the proteins involved in selenoprotein biosynthesis in the willistoni branch. A number of genes seem to have been evolving free of selective constraints, and they cannot be found in the genome of *D. willistoni* (tRNA^Sec^ , *SecS, EFsec*) or they can only be found as residual pseudogenes (*pstk*). These proteins are probably directly involved in selenoprotein biosynthesis, and unlikely to be involved in unrelated functions. A second group of genes (*SPS1* and *Secp43*), in contrast, are as conserved (or almost as conserved) in *D. willistoni* as in the other Drosophila. Selenoprotein loss does not seem to have greatly influenced their evolutionary rate, and they are therefore likely to have additional (and perhaps more important) functional roles not directly related to selenoprotein biosynthesis. SBP2 exhibits a third, intermediate behavior, with the N-terminal region of the protein showing little similarity to the sequence conserved across the Drosophila genomes, but the C-terminal region strongly conserved. This suggests that, while SBP2 plays an important role in selenoprotein biosynthesis, it may also be involved in other unrelated functions.

### Loss of selenoproteins in other insects

In order to get a clearer understanding of the evolution of selenoprotein genes in the class Insecta, we have analyzed the sequences of all published insect genomes: the Diptera *A. gambiae* [23] and *A. aegypti* [24], the Hymenoptera *A. mellifera* (honey bee) [21], and *N. vitripennis* (wasp), the Coleopteron *T. castaneum* (beetle) [22], and the Lepidopteron *B. mori* (silkworm) [25].

Lobanov et al. [20] have recently reported that the species *T. castaneum* and *B. mori* lack selenoproteins. Our results, summarized in Table 2, indicate that in addition to these species and to *D. willistoni*, the Hymenoptera *N. vitripennis* and *A. mellifera* have also lost both selenoproteins and the ability to synthesize them, while mosquitoes maintain the selenoprotein complement of *D. melanogaster*. Like in *D. willistoni*, key components of the selenoprotein machinery have been lost in insects lacking selenoproteins, but not in insects containing them (Table 2). Thus, tRNA^Sec^, *EFsec, pstk*, and *SecS*, which could not be found in *D. willistoni* –and we, therefore, speculated were exclusively involved in selenoprotein biosynthesis–also cannot be found in the genomes of other selenoprotein lacking insects (or they can only be found as pseudogenes: *SecS* in *T. castaneum* and *pstk* in *D. willistoni*). They are, however, present in the selenoprotein coding genomes of the mosquitoes. *SecS*, found in the genome of *A. mellifera* is the only exception to this trend (Figure S6).

**Table 2:** A summary of the results for each selenoprotein and selenoprotein factor in all completely sequenced insect genomes.

	SelH	SelK	SPS2	SPS1	SBP2	EfSec	tRNA^Sec^	Secp43	SecS	pstk
*Drosophila*	+	+*	+	+	+	+	+	+	+	+
***D. willistoni***	Cys	Cys	−	+	+	−	-(?)	+	−	-(?)
*A. gambiae*	+	+	+	+	+	+	+	-(?)	+	+
*A. aegypti*	+	+	+	+	+	+	+	+	+	+
***A. mellifera***	−	−	−	+	+	−	−	+	+	−
***N. vitripennis***	−	−	−	+	+	−	−	+	−	−
***B. mori***	−	−	−	+	−	−	-(?)	+	−	−
***T. castaneum***	−	Cys	−	+	−	−	-(?)	+	-(?)	−

Species lacking selenoproteins are shown in bold. “+” means the gene was present and conserved, “Cys” means the gene was found as a cysteine homolog, “−” means the gene was absent and “-(?)” means that a candidate could be found but the conservation, or score in the case of tRNASec was too low for it to be considered a bona fide homolog.

* Except for *D.persimilis,* see text.

As with *D. willistoni*, the matches found in these genomes for *EFsec* actually correspond to *EFtau*. The results of our search for tRNA^Sec^ (Table 1) are at first glance puzzling: consistent with the observed pattern of presence/absence of selenoproteins, no eukaryotic tRNA^Sec^ could be predicted in the genomes of *A. mellifera*, and *N. vitripennis*, and only a poor one in the genome of *D. willistoni*, while a very strong candidate can be identified in the selenoprotein containing genome of *A. aegypti*. However, relatively strong tRNA^Sec^ predictions are obtained in the genomes of *B. mori* and *T. castaneum*, which lack selenoproteins, while in contrast only a relatively poor prediction is obtained in the selenoprotein containing genome of *A. gambiae*. Close inspection of the predicted tRNA^Sec^ genes, however, shows that the tRNA^Sec^ predicted in *A. gambiae* strongly resembles that of the other selenoprotein containing Diptera, while the sequence of the tRNA^Sec^ predicted in *B. mori, T. castaneum* and *D. willistoni* are very divergent (Figure 6).

*SPS1* and *Secp43*, which were as conserved in *D. willistoni* as in the other Drosophila species–and which we therefore speculated are involved in additional functions not related to selenoprotein biosynthesis–can be found in all investigated species, irrespective of selenoprotein coding capacity. Although we found no good *secp43* candidate in *A. gambiae*, we did find a chimeric match against chromosome 2R and chromosome 3L, suggesting an assembly error. More intriguing is the case of *SPS1* in *A. mellifera*. As with *D. willistoni*, the *D. melanogaster SPS1* and *SPS2* map to the same location in the *A. mellifera* genome and analysis of the sequence alignments strongly suggests that the *A. mellifera* gene is the SPS1 homolog (Figure 4). In contrast with all other insect *SPS1s*, the *A. mellifera SPS1* contains a TGA codon at the position of the conserved Arginine (R) residue, which is orthologous to the Sec encoding TGA in *SPS2*. In addition, *A. mellifera* is the only species of those which we claim lack selenoproteins that retains the selenoprotein specific factor *SecS*. However, only an unstable SECIS (Free energy: -1.20), exhibiting moreover a bulge immediately after the conserved core (Figure S9) can be found in the region immediately 3′ of this gene. In addition, as we have already seen, not only can none of the known fly selenoproteins be found in *A. mellifera* but three of the factors involved in selenoprotein synthesis (*pstk, Efsec* and tRNA^Sec^) are also absent. We believe therefore that the *A. mellifera SPS1* is not a selenoprotein, but that the TGA codon is readthrough by an alternative mechanism to produce the full length SPS1 protein. Stop codon readthrough is not uncommon in *D. melanogaster* [42], [43]. TGA is known to be the most “leaky” STOP codon (e.g. [44]) and has been shown to direct incorporation of Arginine [45]. Structural analysis of the putative *SPS1* mRNA using mfold [46], [47] showed that the TGA is in a region of high structural stability and forms part of a stem-loop (data not shown) both of which have been shown, together with a Guanine residue observed at the position +3 downstream of the TGA codon, to enhance readthrough in *D. melanogaster* [42].

*SBP2*, which was partially conserved in *D. willistoni*, is more confusing when analyzed across all insect genomes. *SBP2* appears to be absent in the selenoprotein lacking genomes of *B. mori* and *T. castaneum*. The conserved C-terminal region is recognizable, however, albeit quite divergent in the genomes of the selenoprotein lacking Hymenoptera. Within Diptera, *SBP2* is present both in mosquitoes and flies. Although no disabling insertion, as in *D. willistoni*, can be found in the SECIS binding domain of other selenoprotein lacking species, overall this domain is slightly more conserved within selenoprotein containing genomes (Figure 7).

**Figure 8:**
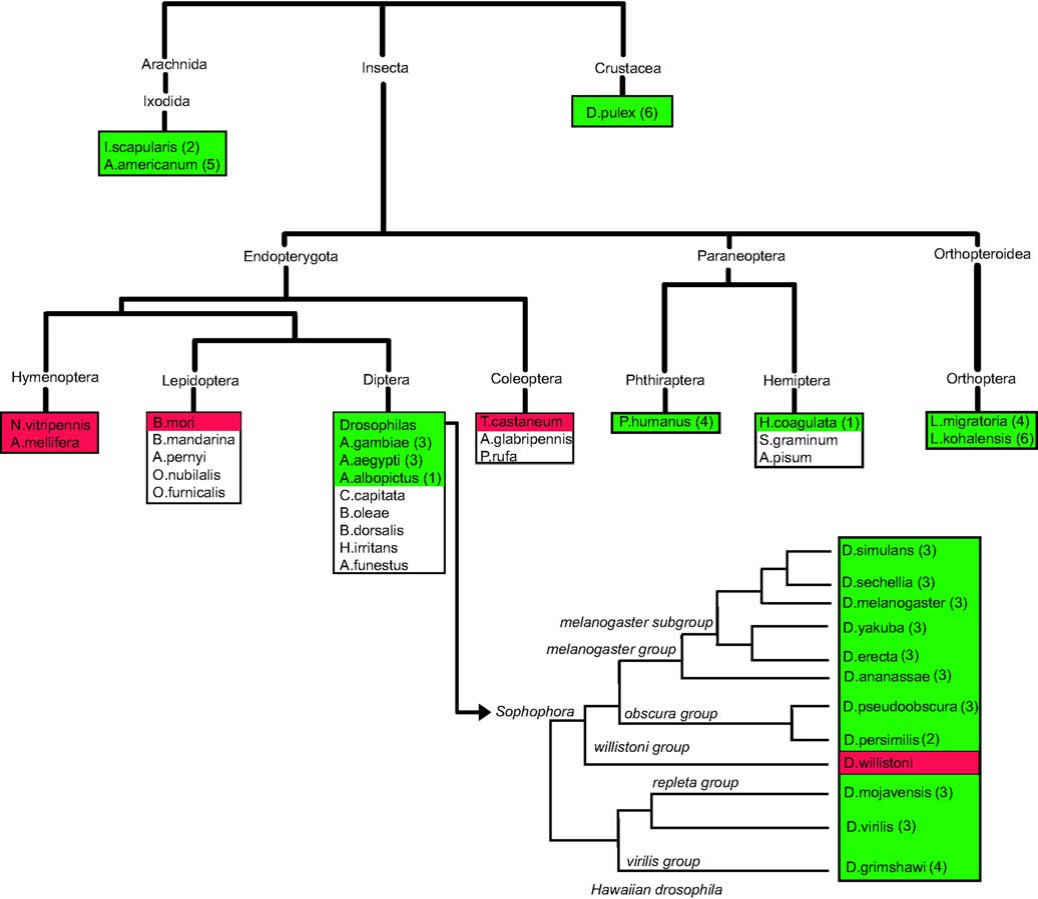
Selenoprotein extinction in arthropoda. Species whose genomes do not code for selenoprotein genes are shown in red. Sec-encoding species are shown in green with the number of selenoproteins found in each genome in parentheses next to its name. Species for which the available data was inconclusive are shown in white. The phylogenetic relationships have been taken from the ncbi's Taxonomy database (http://www.ncbi.nlm.nih.gov/Taxonomy/) and the Tree of Life project (http://www.tolweb.org/tree/). The Drosphilidae tree was taken from the Drosophila Sequencing Consortium wiki (http://rana.lbl.gov/drosophila/caf1.html).

We have extended our analysis by searching genomic, EST, cDNA and peptide sequences available for other arthropod species (including the Arachnids *Ixodes scapularis* and *Amblyomma americanum*, and the Crustacean *Daphnia pulex* ). Results are summarized in Figure 8. Since none of these genomes (except *D. pulex*) is complete, lack of evidence for selenoproteins cannot be taken to indicate total loss of selenoproteins in a given species. Results are interesting, notwithstanding. We have found no evidence of selenoprotein loss in the genomes of any species outside the Endopterygota. Within this infraclass, species of the Hymenoptera, Lepidoptera and Coleoptera orders whose genomes have been completely sequenced do not code for selenoprotein genes, and no evidence of selenoproteins can be found in the partially sequenced species from these orders. In contrast, within Diptera, we found both selenoprotein coding and non-coding species. In summary, existing data do not support selenoprotein loss outside the Endopterygota, but within this infraclass, the loss appears to be generalized across the orders investigated, with the exception of the Diptera.

## Discussion

Until very recently, selenoproteins were believed to be essential for animal life. The discovery that *D. willistoni* [19], *T. castaneum* and *B. mori* [20] have lost the ability to encode selenoproteins has shaken this belief. Here we present a comprehensive analysis of the selenoproteomes of all available insect genomes, and identify two other insect species, *N. vitripennis* and *A. mellifera*, which have also lost selenoproteins. Through our analysis we have been able to reconstruct a broader picture of selenoprotein extinction in insects.

Strong evidence in support of our conclusions comes from the concomitant loss of the factors required for selenoprotein biosynthesis, that we find systematically associated with the loss of selenoproteins (or with their conversion into Cys-homologs, see Table 2). Contradicting our conclusions, however, selenoprotein genes have been reported, after computational analyses, in the genomes of *B. mori* [25] and *A. mellifera* [21], both of which we claim do not code for selenoproteins. The case of *B. mori* is, in our opinion, unquestionable. The genome of this species lacks all factors specifically associated with selenoprotein synthesis: tRNA^Sec^, *EFsec*, *SecS, pstk* and *SBP2*. Moreover, the known fly selenoproteins are either Cys-homologs (*SelK*) or lost (*SelH* and *SPS2* ). Finally, the evidence provided in support of novel selenoprotein genes is rather weak, and lacks experimental verification. We therefore confidently conclude that *B. mori* does not contain selenoprotein genes. The case of *A. mellifera* is more controversial (see Results). However, the absence of many essential components of the selenoprotein biosynthesis machinery and of all known fly selenoproteins in *A. mellifera*, as well as the weak SECIS element predicted in *SPS1*, strongly suggest that SPS1 is not a selenoprotein in *A. mellifera*, and that this species lacks the capacity to synthesize selenoproteins.

The selective loss of selenoproteins in some insect species allows us to investigate the specificity of the functional role attributed to the known selenoprotein factors and even pinpoint the protein regions responsible for such functional specificity (see Results). We have found that three such factors: tRNA^Sec^, *EFsec*, and *pstk*, are either lost or very degenerate in the genomes of species lacking selenoproteins. We conclude, therefore, that the major role of these genes is indeed related to selenoprotein biosynthesis. We believe that SecS also belongs to this group, although this protein is present in *A. mellifera* (Figure S6). *SPS1* and *Secp43*, on the other hand, are present and conserved in all genomes investigated irrespective of whether or not they code for selenoproteins. We believe, therefore, that these genes are likely to play a very important role, unrelated to selenoproteins. This does not necessarily mean that they are not involved in selenoprotein biosynthesis. The relative acceleration of the rate of sequence change observed in *D. willistoni* suggests that this may be the case at least for SPS1 (Figure 5). Finally, our results indicate that, while SBP2's major role is probably related to selenoprotein biosynthesis, it may also have secondary roles that slow or prevent its elimination from the genome of selenoprotein lacking species. Conversely, selenoprotein loss can also be used to identify novel selenoprotein factors. We have systematically searched for genes that are consistently absent in genomes that do not code for selenoproteins but present in those that do. However, the incomplete status of many of the genomes analyzed confounds analysis of the results of such a search, and we have not been able to confidently identify novel candidate selenoprotein factors.

With very few exceptions (*pstk D. willistoni*, and *SecS* in *T. castaneum*), we have not found pseudogenes for most of the lost selenoproteins and selenoprotein factors. Thus, selenoproteins and selenoprotein factors appear to be either present in a given genome as functional proteins or totally absent (without even a recognizable fossil sequence relic). Certainly, the large phylogenetic distance separating many of the species we have investigated confounds the identification of orthologous genomic regions evolving for extended evolutionary times free of selective constraints. The availability of the genomes of the twelve drosophila species [19], however, allows us to pinpoint the syntenic regions in *D. willistoni* corresponding to the regions that, in the other Drosophila genomes, contain the selenoprotein factors. Thus, for tRNA^Sec^, *EFsec, pstk* and *SecS* —all lost in *D. willistoni* —we have been able to identify the syntenic regions in the genome of this species (see http://genome.imim.es/datasets/2008selenoinsects/#4). For all factors but *pstk*, a deletion in the genome of *D. willistoni* has eliminated the syntenic region, but the upstream and downstream genes can still be found. In the case of *SecS*, the deletion also includes the gene downstream. The fate of SPS2 is harder to determine, since we have not been able to find the syntenic region in *D. willistoni*. It appears therefore, that during Drosophila evolution the entire deletion of non-functional regions and genes is more common—at least in selenoprotein associated genes—than sequence degradation and consequent pseudogenisation. Such behavior is consistent with the dynamic nature of genome micro-structure and the high rate of genome rearrangements observed in Drosophila [19].

Our data strongly suggest that selenoprotein loss has been confined to the Endopterygota within a general trend of reduction in selenoprotein number in this group compared with other insects (Figure 8). Endopterygota are among the most diverse group of insects, comprising eleven orders. Complete genome sequences are only available for species from the orders Coleoptera, Hymenoptera, Lepidoptera, and Diptera. While certainly insufficient for definitive conclusions, available data suggest that selenoprotein loss may be general within Hymenoptera, Coleoptera, and Lepidoptera (all species from these orders with completely sequenced genomes lack selenoproteins and many of the necessary selenoprotein factors, and no evidence of selenoproteins or selenoprotein factors can be found in the species with only partial sequence data available, see Figure 8). In the Diptera, in contrast, only the genome of *D. willistoni*, out of the 14 so far sequenced, has lost selenoproteins. While the phylogenetic relationship between the different orders of this group remain controversial (e.g. [49]–[51]), the selective selenoprotein loss that occurred within the Diptera rules out a single evolutionary event as the origin of the pattern of selenoprotein extinction observed in this group. We believe that this pattern is more consistent with a relaxation in insects— accentuated in the Endopterygota— of the selective constraints to maintain selenoproteins that appear to be acting across metazoans (S. Castellano, pers. comm.). In this scenario, different Endopterygota species would be losing selenoproteins independently. Within this general trend of selenoprotein extinction, punctual events of selenoprotein expansion are still possible, such as the duplication of *SelH* observed in *D. grimshawi.*

Lack of intermediate pseudogene evidence confounds the investigation of the evolutionary events that lead to selenoprotein extinction within the Endopterygota. Nevertheless, two contrasting hypotheses can be formulated. One possibility is that selenoprotein genes are lost (or converted to Cys-homologs) first and this triggers the loss of the selenoprotein factors—by rendering these genes functionally irrelevant and easing the selective constraints acting upon then. The alternative hypothesis is that a disabling mutation in a selenoprotein factor occurs first. This renders the mutated species incapable of selenoprotein biosynthesis, and triggers the conversion of selenoproteins into Cys-homologs or simply their elimination from the genome. Primary loss of selenoproteins appears to be more consistent with the existing Drosophila data. Indeed, *D. persimilis* has lost *SelK* as a selenoprotein and, with the exception of the selenoprotein factor *SPS2*, retains only *SelH* as a selenoprotein. Since *SelH* has a Cys-homolog in *D. persimilis*, the selective constraints acting to maintain the selenoprotein factors, which may already be weak in the Drosophila, may be almost non-existent in this species. The alternative hypothesis cannot be discarded, however, because experimental data exists showing that mutant flies for *EFsec*, which fail to decode TGA as Sec, are viable and fertile [18]. The initial mutation of a selenoprotein factor would therefore not necessarily affect the survival of a species survival, but it would trigger selenoprotein loss. In fact, independent events of selenoprotein extinction may have occurred through different evolutionary paths.

Whatever the path leading to selenoprotein loss, our data indicate that, in contrast to all other known metazoans, selenoproteins and selenoprotein synthesis are dispensable in Drosophila and apparently in the entire Endopterygota infraclass. This agrees with the aforementioned data showing viability of mutant *EFsec* flies [18]. We also think our hypothesis is consistent with the data by Alsina et al. showing that mutant flies for *SPS1* do not contain selenoproteins and are lethal at the third larval instar [17]. Indeed, our data indicates that one of the major roles of SPS1 is likely to be unrelated to selenoprotein biosynthesis (see Results). Therefore, lethality in mutant *SPS1* flies is probably being induced through the impairment of this major role, and not through its effect on selenoprotein biosynthesis. More difficult to reconcile with our hypothesis of dispensability of selenoproteins in Endopterygota are the experiments in which inhibition of either SelK or SelH expression significantly reduces viability in *D. melanogaster* embryos [14], [52]. The possibility exists, however, that the phenotypes observed in the affected flies result from off-target effects of the RNAi molecules used to interfere with *SelH* and *SelK*.

Why the selective constraints acting on selenoproteins have relaxed in Endopterygota remains unknown. However, it is tempting to speculate that they are related to the differences in antioxidant defense systems between Drosophila (and likely other insects) and other metazoans [53]–[56]. Among other differences, flies do not utilize glutathione peroxidases and have replaced glutathione reductase with non-selenoprotein thioredoxin reductases to reduce glutathione [53]. The components of the oxidative stress defense system in insects may have thus become independent of selenoproteins, rendering selenoproteins (which in vertebrates have well-established anti-oxidant and cellular redox functions) dispensable. This hypothesis is supported by a recent comparison of the antioxidant defense systems of *A. mellifera, A. gambiae* and *D. melanogaster* [57] that showed a differential expansion of antioxidant gene families between the Sec-lacking *A. mellifera and* the two Sec-encoding Diptera.

Selenoproteins, on the other hand, may also play a functional role in metazoans by sequestering selenium. Sequestration of selenium, whose excess in diet is highly toxic, would thus be compromised in the selenoprotein lacking Endopterygota. We have investigated other non-selenoprotein selenium binding proteins, but have found no signature specific to selenoprotein-lacking insects. How these animals deal with excess selenium represents, therefore, an avenue of future research. In general, that some animals can live without selenoproteins should contribute to a better understanding of the functional role and evolutionary history of this intriguing family of proteins, the most striking exception to the universality of the genetic code.

## Supporting Information

Figure S1 Alignment of insect SelH SECIS elements. Alignment of predicted SelH SECIS elements from each of the insects investigated in which the gene was found. Magenta boxes bound the conserved regions of the SECIS element.
(0.64 MB DOC)

Figure S2 Phylogenetic tree built from the alignment of SelK and SelK cysteine paralogs. Phylogenetic tree built from an alignment of SelK and SelK cysteine paralogs (identified with a “C” after the name of the species) across the 12 Drosophila and *A. Gambiae* (used as an outgroup to root the tree). *D. willistoni* is shown in magenta.
(0.05 MB TIF)

Figure S3 Alignment of insect SelK SECIS elements. Alignment of predicted SelK SECIS elements from each of the each of the insects investigated in which the gene was found. Magenta boxes bound the conserved regions of the SECIS element. Note the loss of conservation in the *D. persimilis* and *D.willistoni* fossil SECIS elements.
(0.65 MB DOC)

Figure S4 SelK cDNA alignment across the 12 Drosophila. Only the terminal region upstream of the stop codon (the last codon in the alignment) is shown. The arrows point to the inserted “T” which has caused a frameshift in *D. persimilis* and the selenocysteine codon (TGA). See Figure 3 for the effect of the frameshift on the protein sequence of SelK in *D. persimilis.*
(0.55 MB DOC)

Figure S5 EFsec alignment across all insects investigated.
(2.41 MB TIF)

Figure S6 SecS alignment across all insects investigated.
(2.93 MB TIF)

Figure S7 PSTK alignment across all insects investigated.
(1.72 MB TIF)

Figure S8 secp43 alignment across all insects investigated.
(3.27 MB DOC)

Figure S9 *A. mellifera* SPS1 potential SECIS element. Potential SECIS element 3′ of *A. mellifera* SPS1 gene using the program SECISearch (free energy: -1.20).
(0.06 MB DOC)
