# Correction: Relaxation of Selective Constraints Causes Independent Selenoprotein Extinction in Insect Genomes

**DOI:** 10.1371/annotation/93ab40c4-c4b0-4f77-a18f-804b74825fb7

**Published:** 2009-07-10

**Authors:** Charles E. Chapple, Roderic Guigó

The file extensions for Figures S1, S3, S4, S8, and S9 are incorrect. They are all TIFF files and can be viewed using the following steps:

1. Click on the link and select "Save to Disk" to save the file to your computer.

2. Change the filename extension from ".doc" to ".tif".

3. Select any program capable of viewing and/or editing images to review the figure.

