# Supplementary figures and images for: Relaxation of Selective Constraints Causes Independent Selenoprotein Extinction in Insect Genomes
Source: PLoS One. 2008 Aug 13;3(8):e2968. doi: 10.1371/journal.pone.0002968 (PMC2500217; doi:10.1371/journal.pone.0002968)

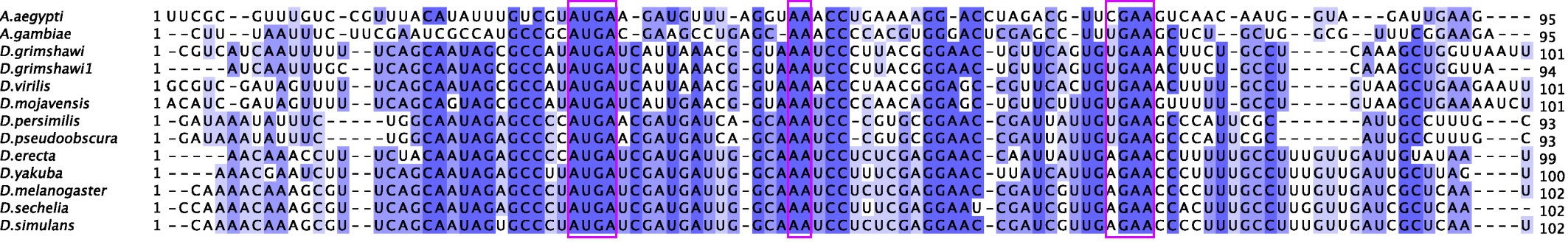

Supplement: Figure S1 — Alignment of insect SelH SECIS elements. Alignment of predicted SelH SECIS elements from each of the insects investigated in which the gene was found. Magenta boxes bound the conserved regions of the SECIS element. (0.64 MB DOC) [file pone.0002968.s001.doc]

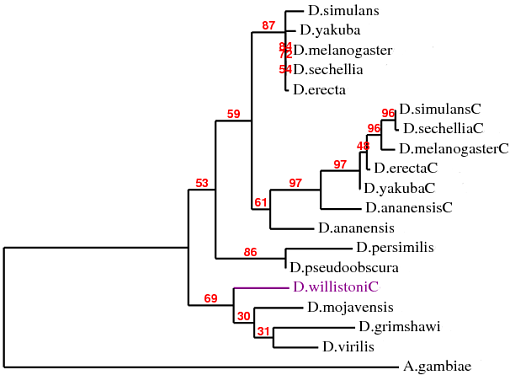

Supplement: Figure S2 — Phylogenetic tree built from the alignment of SelK and SelK cysteine paralogs. Phylogenetic tree built from an alignment of SelK and SelK cysteine paralogs (identified with a “C” after the name of the species) across the 12 Drosophila and A. Gambiae (used as an outgroup to root the tree). D. willistoni is shown in magenta. (0.05 MB TIF) [file pone.0002968.s002.tif]

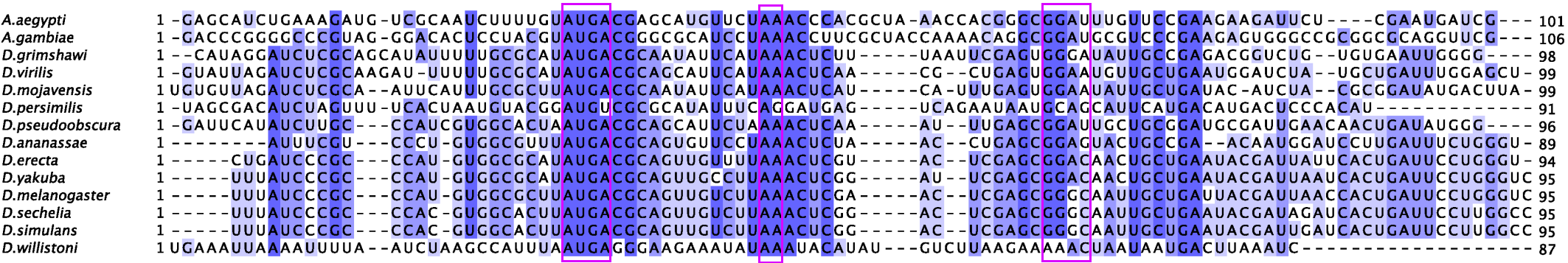

Supplement: Figure S3 — Alignment of insect SelK SECIS elements. Alignment of predicted SelK SECIS elements from each of the each of the insects investigated in which the gene was found. Magenta boxes bound the conserved regions of the SECIS element. Note the loss of conservation in the D. persimilis and D.willistoni fossil SECIS elements. (0.65 MB DOC) [file pone.0002968.s003.doc]

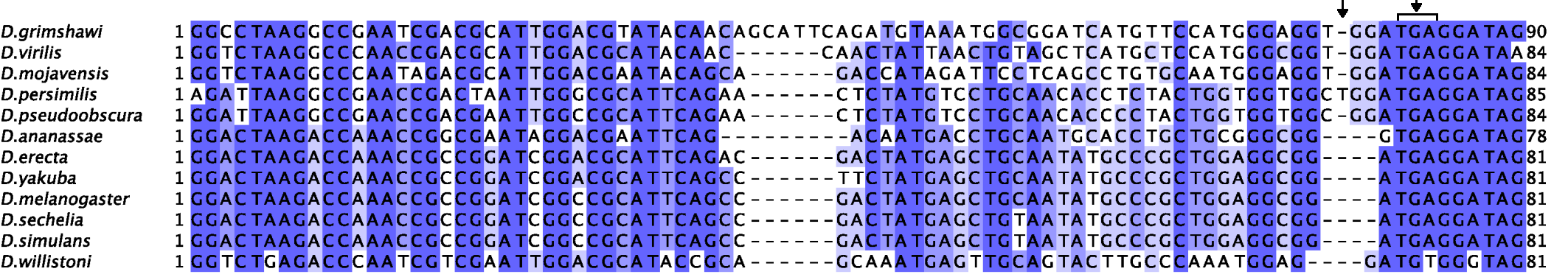

Supplement: Figure S4 — SelK cDNA alignment across the 12 Drosophila. Only the terminal region upstream of the stop codon (the last codon in the alignment) is shown. The arrows point to the inserted “T” which has caused a frameshift in D. persimilis and the selenocysteine codon (TGA). See Figure 3 for the effect of the frameshift on the protein sequence of SelK in D. persimilis. (0.55 MB DOC) [file pone.0002968.s004.doc]

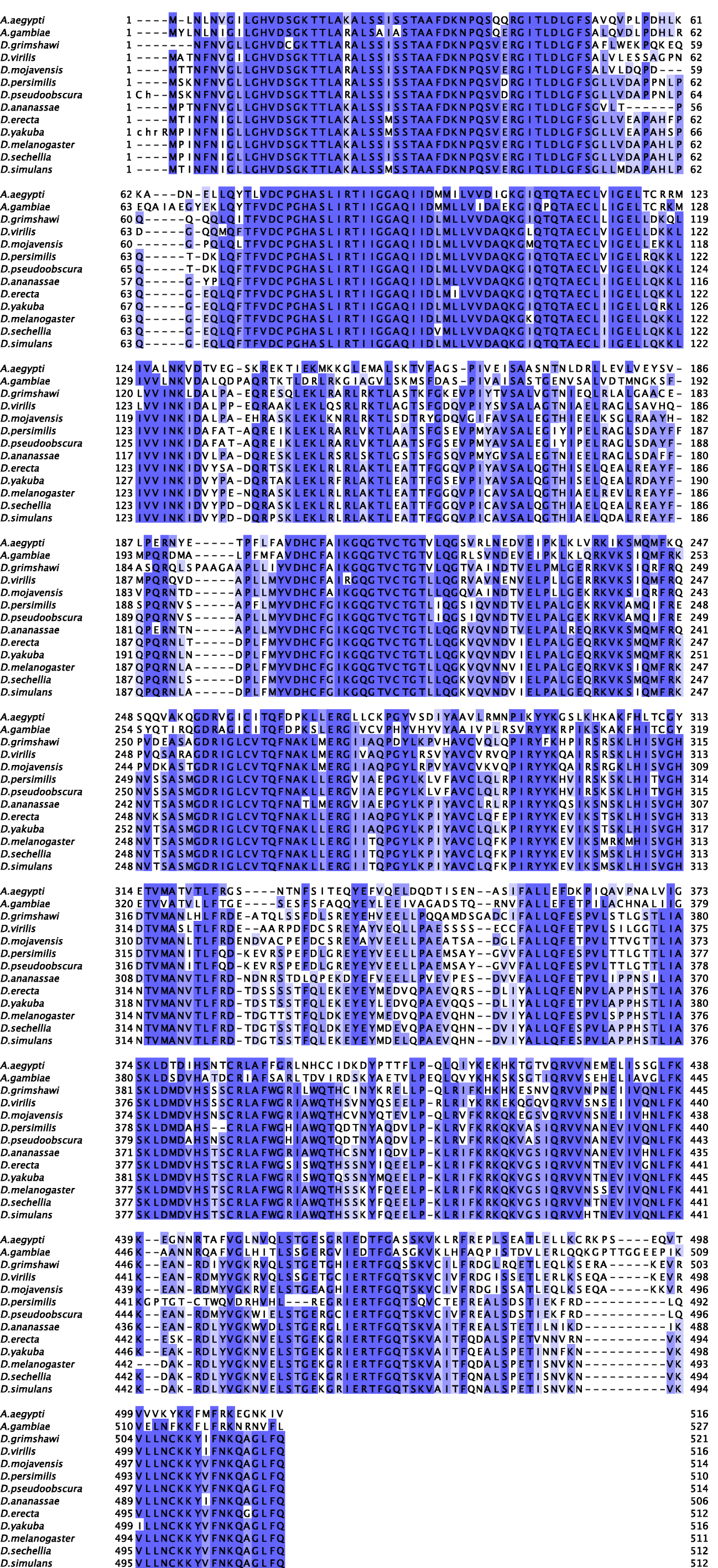

Supplement: Figure S5 — EFsec alignment across all insects investigated. (2.41 MB TIF) [file pone.0002968.s005.tif]

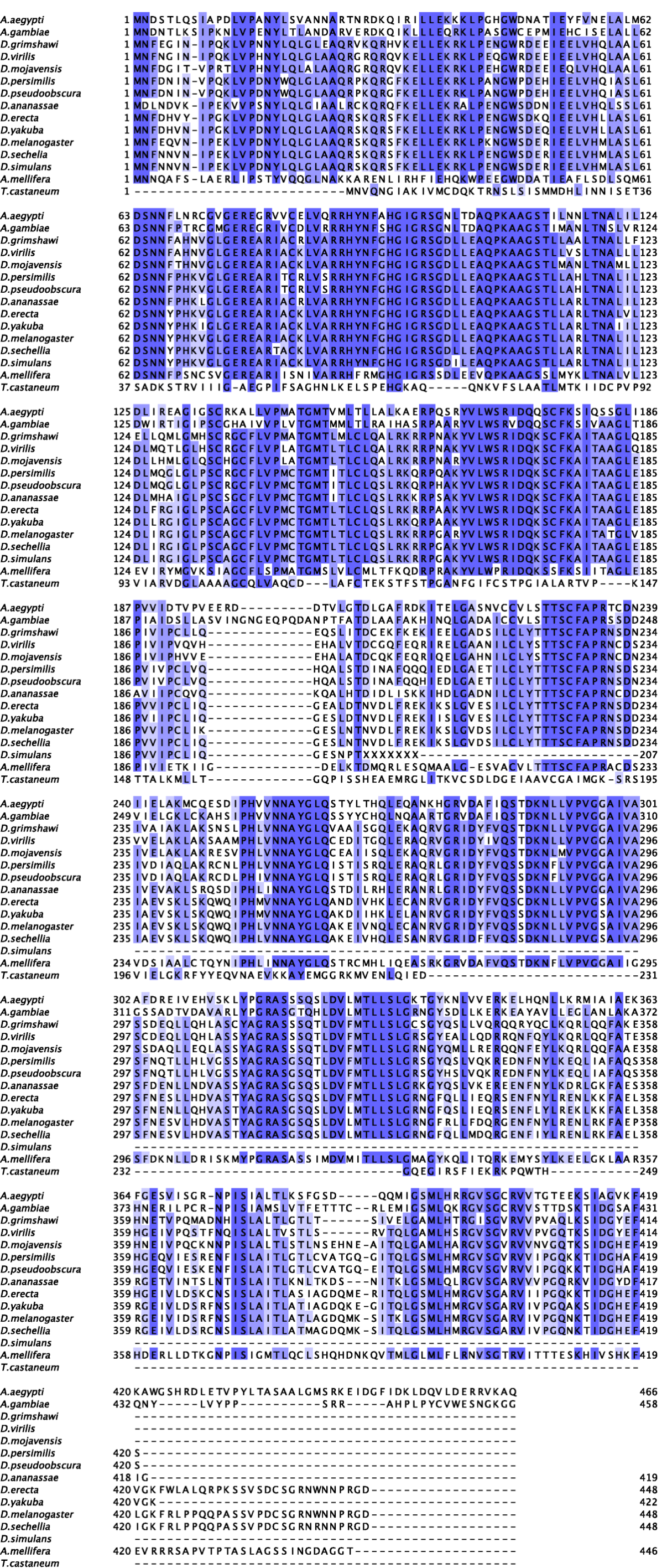

Supplement: Figure S6 — SecS alignment across all insects investigated. (2.93 MB TIF) [file pone.0002968.s006.tif]

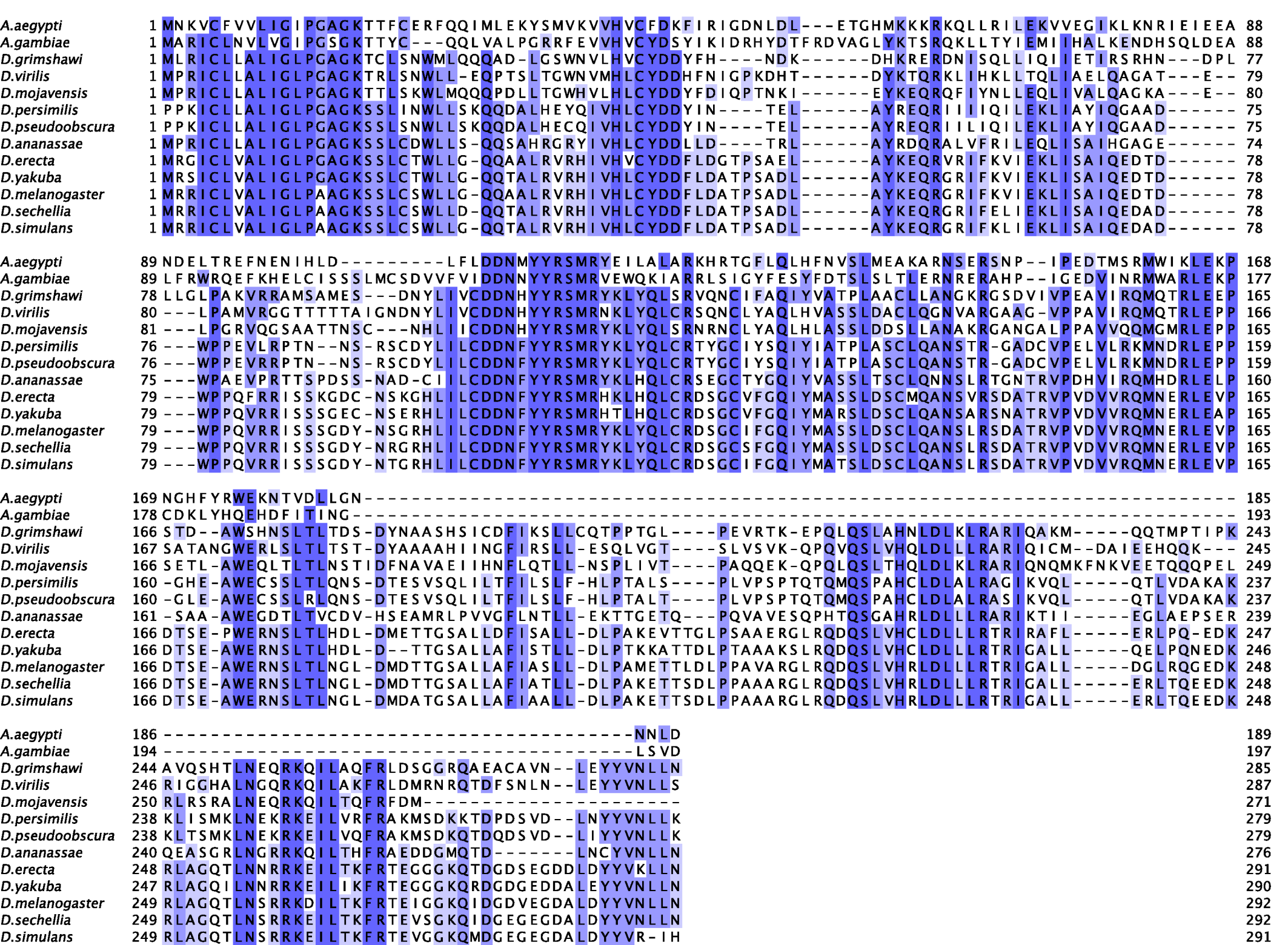

Supplement: Figure S7 — PSTK alignment across all insects investigated. (1.72 MB TIF) [file pone.0002968.s007.tif]

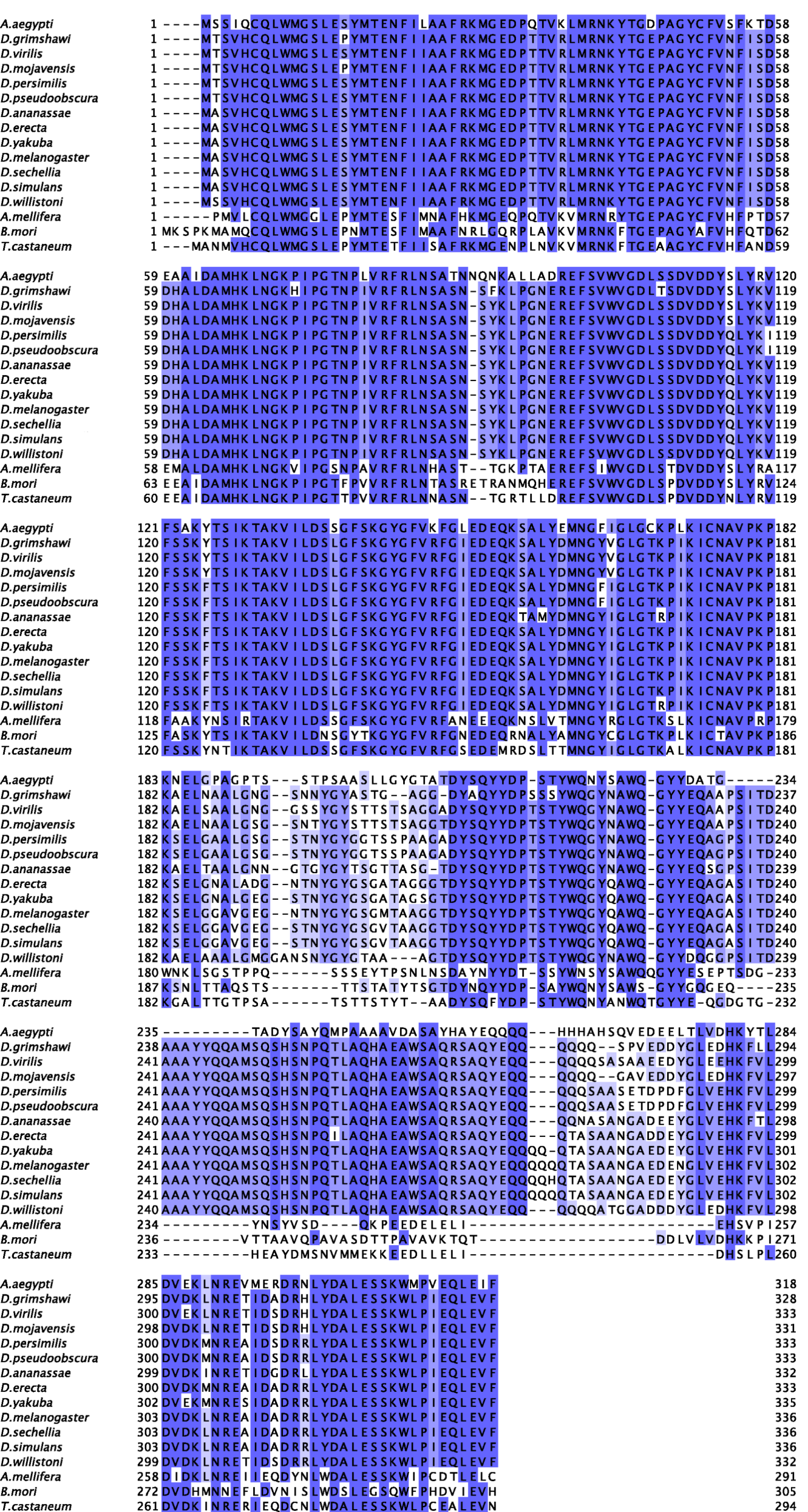

Supplement: Figure S8 — secp43 alignment across all insects investigated. (3.27 MB DOC) [file pone.0002968.s008.doc]
